# Supplementary material for: Blood RNA biomarkers and a point-of-care elastase assay for detecting host immune activation in suspected sepsis: Trajectory matters
Source: PLoS One. 2025 Dec 12;20(12):e0338012. doi: 10.1371/journal.pone.0338012 (PMC12700442; doi:10.1371/journal.pone.0338012)
Supplement: S1 Table — (DOCX) [file pone.0338012.s001.docx]

| **Supplemental Table 1. Demographic Values for ED and Control Groups** | | | | | |  |  |  |  |
| --- | --- | --- | --- | --- | --- | --- | --- | --- | --- |
|  | **ED** | **SEM** |  | **Non-ED** | **SEM** |  | **Non-ED** | **SEM** |  |
|  | **Patients** |  |  | **Control** |  |  | **Infection** |  |  |
| **N** | 72 | - |  | 16 | - |  | 8 | - |  |
| **Mean Age (yrs)** | 50.99 | 2.19 |  | 32.88 | 3.91 | * | 35.63 | 6.48 | * |
| **Sex (% male)** | 58.33 | - |  | 81.25 | - |  | 62.50 | - |  |
| **Race** |  |  |  |  |  | * |  |  | * |
| **% White** | 31.94 | - |  | 93.75 | - |  | 75.00 | - |  |
| **% Black** | 61.11 | - |  | 0.00 | - |  | 0.00 | - |  |
| **% Other** | 6.94 | - |  | 6.67 | - |  | 0.00 | - |  |
| **Ethnicity (% non-Hispanic)** | 91.55 | - |  | 100.00 | - |  | 87.5 | - |  |

**Notes:** Percent values calculated using the total number of patients with available data for each variable as the denominator. Data were available for all patients across all variables except two patients with unknown ethnicity. Asterisks indicate statistically significant differences between the ED vs. control groups based on p-values < 0.05 using equal variance two-tailed t-tests for continuous variables and two-tailed Fisher's Exact Tests for categorical variables. The Fisher's Exact Test for the race variables is for white vs. non-white participants. The Fisher's Exact Test for the ethnicity variable combines both control groups to avoid categories where N=0. ED = Emergency Department. SEM = Standard Error of the Mean.

**For comparison purposes, from the main manuscript:**

| **Table 1. Demographic and Lab Values** | |  |  |  |  |  |  |  |
| --- | --- | --- | --- | --- | --- | --- | --- | --- |
|  | **SEPSIS** | **SEM** |  | **SEPSIS** | **SEM** |  | **NO** | **SEM** |
|  | **/SHOCK** |  |  |  |  |  | **INFECTION** |  |
| **N** | 22 | - |  | 26 | - |  | 24 | - |
| **Age (yrs)** | 57.95 | 3.73 | * | 47.19 | 3.93 |  | 48.71 | 4.38 |
| **Weight (kg)** | 88.28 | 5.73 |  | 84.56 | 6.77 |  | 94.91 | 6.34 |
| **Sex (% male)^†^** | 36.40 | - |  | 50.00 | - |  | 37.50 | - |
| **Race (% white)^†^** | 27.27 | - |  | 38.46 | - |  | 29.17 | - |
| **Body Temperature (F)** | 99.73 | 0.50 | * | 98.85 | 0.37 |  | 98.40 | 0.35 |
| **Systolic Blood Pressure (mmHg)** | 129.77 | 9.00 |  | 131.88 | 4.95 | * | 146.52 | 5.72 |
| **Diastolic Blood Pressure (mmHg)** | 75.86 | 4.39 |  | 77.40 | 3.16 |  | 85.91 | 4.78 |
| **Heart Rate (beats per minute)** | 118.45 | 5.84 |  | 105.32 | 4.11 |  | 114.52 | 4.01 |
| **Respiratory Rate (breaths per minute)** | 23.76 | 1.85 |  | 20.05 | 1.13 |  | 22.05 | 1.70 |
| **White Blood Cells (K/µL)** | 14.87 | 1.38 |  | 13.46 | 1.11 |  | 12.36 | 1.91 |
| **Red Blood Cells (M/µL)** | 4.13 | 0.23 |  | 4.04 | 0.25 |  | 3.93 | 0.18 |
| **Hemoglobin (g/dL)** | 12.24 | 0.72 |  | 11.73 | 0.67 |  | 11.21 | 0.56 |
| **Hematocrit (% packed RBC)** | 37.50 | 1.98 |  | 36.05 | 2.00 |  | 35.26 | 1.59 |
| **Mean Corpuscular Volume (fL)** | 91.40 | 1.87 |  | 90.18 | 1.57 |  | 90.51 | 2.35 |
| **Mean Corpuscular Hemoglobin (pg)** | 29.60 | 0.66 |  | 29.27 | 0.59 |  | 28.77 | 0.98 |
| **MCH Concentration (g/dL)** | 32.41 | 0.37 |  | 32.46 | 0.39 |  | 31.68 | 0.49 |
| **Red Cell Distribution Width (mean %)** | 15.37 | 0.68 |  | 14.53 | 0.46 |  | 15.47 | 0.59 |
| **Platelet Count (K/µL)** | 260.52 | 38.89 |  | 327.62 | 42.27 | * | 222.21 | 35.96 |
| **Bands (%)** | 5.75 | 2.37 |  | 0.27 | 0.20 |  | 0.33 | 0.22 |
| **Neutrophils (%)** | 76.80 | 2.79 | * | 78.96 | 1.65 | * | 64.43 | 4.37 |
| **Lymphocytes (%)** | 13.50 | 2.46 |  | 11.46 | 1.19 | * | 22.13 | 3.45 |
| **Monocytes (%)** | 7.47 | 0.80 |  | 7.67 | 0.56 |  | 8.61 | 1.06 |
| **Eosinophils (%)** | 0.38 | 0.15 | * | 0.88 | 0.33 |  | 1.73 | 0.53 |
| **Basophils (%)** | 0.07 | 0.06 |  | 0.08 | 0.06 | * | 0.45 | 0.18 |
| **Immature Granulocytes (%)** | 0.85 | 0.13 | * | 0.62 | 0.13 |  | 0.40 | 0.03 |
| **Urine Leukocyte Esterase (% +)^‡^** | 50.00 | - | * | 25.00 | - |  | 7.69 | 0.19 |
| **Urine Nitrate (% +)^‡^** | 22.22 | - |  | 16.67 | - |  | 0.00 | - |
| **Urine White Blood Cells (% +)^†^** | 76.19 | - | * | 41.67 | - |  | 39.13 | - |
| **Urine White Blood Cell Count** | 191.06 | 95.31 |  | 41.70 | 13.99 |  | 1.78 | 0.33 |
| **Blood Culture (% +)^‡^** | 40.00 | - | * | 5.00 | - |  | 0.00 | - |
| **C-reactive Protein (mg/L)** | 198.56 | 35.32 |  | 146.76 | 22.51 |  | 186.73 | 28.05 |
| **Lactic Acid (mmol/L)** | 3.59 | 0.51 |  | 1.88 | 0.18 |  | 3.13 | 0.82 |

Notes: Values are means unless otherwise indicated. Percent values calculated using the total number of patients with available data for each variable as the denominator. Asterisks indicate statistically significant differences between the severe sepsis vs. sepsis and/or no infection groups and the sepsis vs. no infection groups based on p-values < 0.05 using equal variance two-tailed t-tests for continuous variables and either chi-squared tests or Fisher’s Exact Tests for categorical variables based on sample size. SEM = Standard Error of the Mean.

^†^ Chi-squared test

^‡^ Fisher’s Exact Test (note: no infection group excluded in the blood culture and urine nitrate tests to avoid categories where N=0)
