# Supplementary material for: Blood RNA biomarkers and a point-of-care elastase assay for detecting host immune activation in suspected sepsis: Trajectory matters
Source: PLoS One. 2025 Dec 12;20(12):e0338012. doi: 10.1371/journal.pone.0338012 (PMC12700442; doi:10.1371/journal.pone.0338012)
Supplement: S1 Fig — (DOCX) [file pone.0338012.s003.docx]

**Supplementary Figure 1.** Temporal changes in RNA biomarkers grouped by Sepsis-3 Criteria.

**
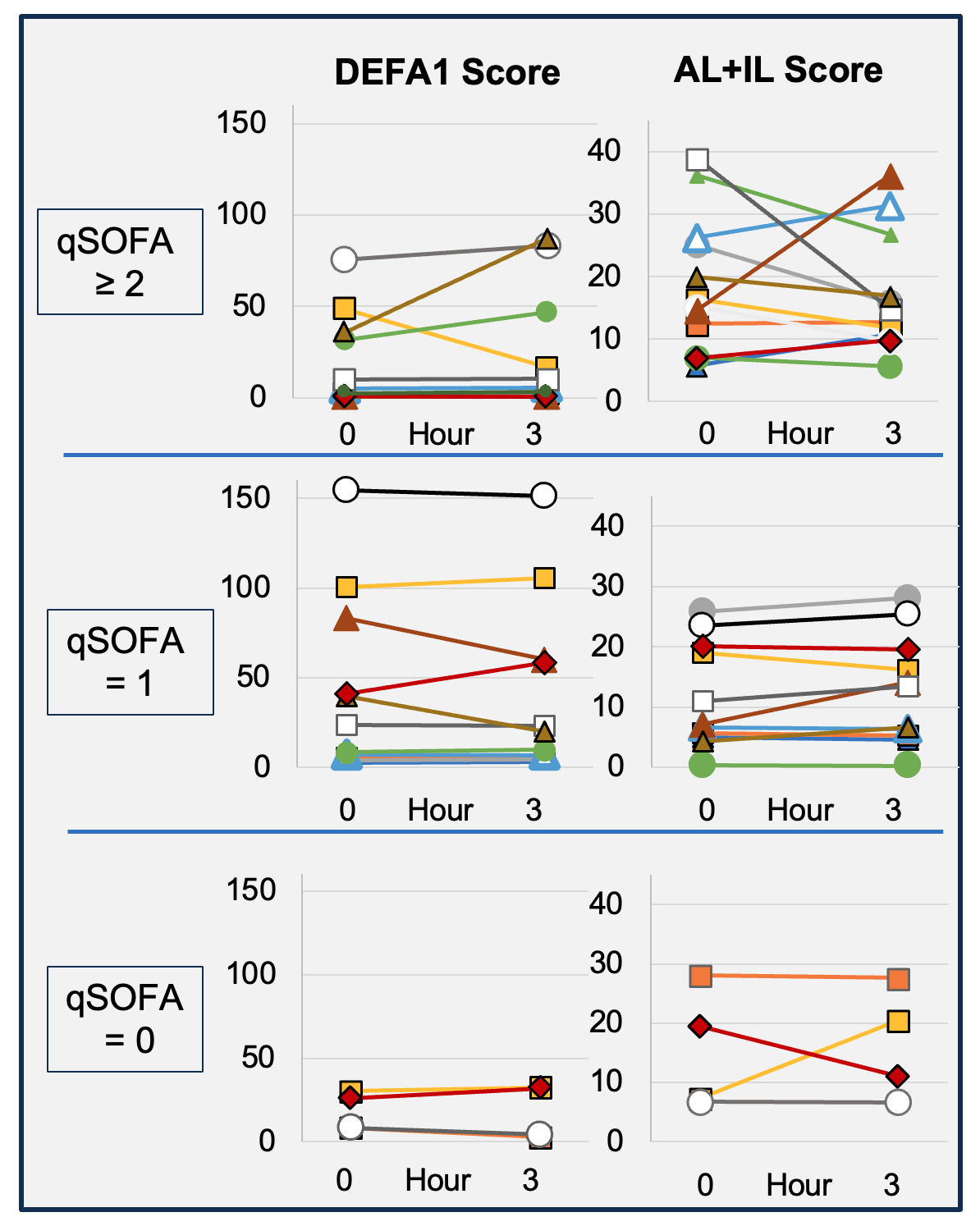
**

**Supplementary Figure 1. Temporal changes in host immune biomarkers associated with Sepsis-3-related qSOFA scores**. A subset of patients were enrolled in which 0 hr and 3 hr samples were obtained. Each line reflects the same patient at two time points for bacterial response markers: DEFA1 RNA, (left panels), and ALPL+IL8RB/CXCR2 (right panels). The panels show patients with qSOFA scores 2 or greater, (n=12), qSOFA scores of 1 (n=11), and qSOFA scores of 0 (n=4).
