# Supplementary material for: Blood RNA biomarkers and a point-of-care elastase assay for detecting host immune activation in suspected sepsis: Trajectory matters
Source: PLoS One. 2025 Dec 12;20(12):e0338012. doi: 10.1371/journal.pone.0338012 (PMC12700442; doi:10.1371/journal.pone.0338012)
Supplement: Attachment — Submitted filename: 0 SENSOR Response to Reviewers.docx [file pone.0338012.s005.docx]

**Response to Critique: LaFleur et al. Blood RNA biomarkers and a point-of-care elastase assay for detecting host immune activation in suspected sepsis: Trajectory matters.**

**Reviewer #1:**

We are very grateful for your thoughtful review, which is constructive, detailed, and accurate. We believe that all of the concerns are addressed to the best of our abilities, and that the manuscript is improved because of the changes. We include a ‘track changes’ version so that it is easy to identify the extensive revisions, including new data analysis, that were made to address these concerns.

**Introduction:**

**Concern 1:** The discussion regarding the treatment of sepsis, while true, is largely unnecessary and impertinent to the current discussion, which is one of diagnosis (not treatment).

**Response:** We have shortened this section to discuss only how diagnostic uncertainty drives needless treatments.

**Concern 2:** The results are ‘teased’ in the introduction, which is somewhat unusual and should be reserved for the results and discussion sections.

**Response:** We have also shortened this section to simply frame the question, without mentioning the outcomes.

**Methods:**

**Concern 3:** The authors point to the criteria used in the sepsis alert; however, these criteria are not clear. Please include the referenced figure (i.e., are these the criteria noted as ‘enrollment criteria’ in figure 1?
In using these criteria, it should be noted that these are not SIRS criteria, but they are a modified version of SIRS. For example, the Temp > 38.3 is higher than traditional SIRS criteria, as are both the Respiratory and Heart Rates. In this way, this modification of SIRS criteria will be less sensitive than previously noted; it is unclear the impact that the glucose will have. In essence, this will result in an included population that is somewhat different than any current definition of sepsis, and may impact the generalizability of the results.

**Response:** These are valid points. Yes, the values in Figure 1 are the criteria used for the Cerner ‘sepsis alert’ that identified subjects for screening and consent. The Cerner alert is based on the St. John’s sepsis alert, which is now cited for clarity and fully detailed in Methods. While it is slightly different than the SIRS criteria, it is a standardized system that many ED physicians are familiar with. We corrected all ‘SIRS criteria’ to ‘sepsis alert’, which is clearly defined as a modified SIRS criteria in the methods. Further, toward a similar point discussed later, we went back to the medical records and computed qSOFA scores used in the SEPSIS-3 criteria (see below).

**Concern 4:** How was the subset of patients and controls selected for measurement of the defensin-α1 level selected? Was this selection random, or was it based on specific criteria, or perhaps through more practical reasons (availability of sample for testing).

**Response:** Yes, the selection of samples for defensin-a1 protein level was made purely on the basis of having plasma available for testing. This is now noted in Methods.

**Concern 5:** With regards to controls … in many cases, understanding the control group becomes one of the most integral components to interpreting a study. There is mention that a normal control group was derived from medical center personnel, and, presumably, another group of subjects with self-reported infections was enrolled. How were these subjects selected? Who are they? A description is important because a 25 year-old medical resident is vastly different than a 65 year old ED patient; the mechanism of selection of the control group is important to disclose. The control group is not included in any description. They should be included in Table 1, if not elsewhere. It is possible that they are in an original format, but, for the PDF available for review, I do not see a column for controls. There is some mention of ‘non-ED controls with self-reported infections’ in the methods; however, there is no detail in how these controls were selected. More clarity is necessary as mentioned in the above comments about the methods.

**Response:** The control group, including those with self-reported infections, are now more fully described in the text. We prepared summary statistics of age, sex, and race, and this is included as Supplementary Table 1. It would not be practical to include the controls in Table 1 because there are no lab values available on these non-patient controls. However, we have repeated the other groups in the supplementary table so that they can easily be compared. In short, the controls are younger and more likely to be White, but their main purpose is simply to provide a reference for non-ED patients, with or without self-reported infection, and they fulfill that need. We extensively revised Table 1 to include full titles for the variables, with additional details in the legend.

**Results:**

**Concern 6:** Overall, the number of groups included in the study is somewhat difficult to follow (there are 5 – control, self-reported infection, no sepsis, sepsis, septic shock). There may be a way to simplify this, and the N’s of the control groups are small enough that it is unclear that they provide any meaningful information.

**Response:** There are 16 controls without any self-reported infection, and this adds a valuable frame of reference for the 8 sub-clinical infections, and the total of 72 ED patients. The n is sufficient to detect statistically significant differences in the RNA biomarker levels (Figure 2). Based on the new Sepsis-3 analysis, we provide new groupings that are simpler, and more amenable to the terminology moving forward.

**Concern 7:** The first paragraph of the results is the first mention of adjudication for sepsis and sepsis shock. Because there is no reference standard for sepsis, it is necessary to have such a process. This does raise several questions: how was the adjudication performed? Why did the authors choose to use the now antiquated terminology of ‘sepsis / severe sepsis / septic shock,’ which were abandoned in the current Sepsis-3 definition referenced in the Introduction. Also, as previously noted, the vital sign criteria are modified from the original SIRS criteria such that these patients may or may not meet any particular definition of sepsis. An explanation for these criteria and a summary of the adjudication process should be included in the Methods. […] Again, a more detailed explanation or description of the adjudication process would be appropriate. It is difficult to interpret inter-observer variability for infection and sepsis without understanding the process by which the determination was made, especially since this definition of sepsis is not consistent with any of the prior definitions that have been published.

**Response:** True, we used the prior Sepsis-2 terms because those are still terms relevant to billing in many US centers, and are still very familiar to most practicing physicians. We agree that Sepsis-3 terms will be more relevant moving forward, and so we reanalyzed the medical records to build the Sepsis-3 qSOFA scores and we present similar results using this new analysis.

The adjudication process is now explained in greater detail, and it is supplemented by the new analysis using the Sepsis-3 criteria to group the patients. We think that with the clarification of the Cerner Sepsis alert, the clarified adjudication process, and the new qSOFA analysis that readers will be able to understand the patient groups adequately. We show the results of the qSOFA analysis as new panels in Figure 2, and as a new Supplementary Figure 1 for the trajectories. Further, to clarify the group names in this analysis, we changed “Severe Sepsis” to “Sepsis/Shock” to indicate that this group is more severe sepsis and includes patients with organ failure signs and vasopressors in some cases.

**Concern 8:** “Average lactate levels were artificially high …” This statement is incorrect; there is nothing ‘artificial’ about the lactic acidosis associated with such physiologic derangements as alcohol withdrawal and seizure.

**Response:** Apologies, we meant that the elevated lactate was not related to infection. We have corrected the wording to “higher than expected”.

**Concern 9:** Table 1: This table needs a legend. Are these means, medians? It should also be reformatted to read easier. Blood culture (+) – is this percent? Presumably so – but is this percent of cultures drawn or percent of patients? We could presume that all patients had blood cultures drawn, but this presumption may not be accurate. Where appropriate, units should be included. I note that, in race, the %white is on average, about 33%. Since this represents a minority of the study population, it would be helpful to have more information on the ethnic and racial distribution. Also, the number of patients in each group is important, given than many of the patients are unlikely to have had specific values tested (CRP, lactic acid, etc), given the data were derived from standard care.

**Response:** Table 1 has been completely revised with the variables spelled out and a legend that clearly denotes that they are means, sems, and how the statistics were conducted.

**Concern 10:** The term “simple sepsis” is used. Colloquially, this is often the term we use for ‘SIRS with a source.’ This is not a technical term, and it should be defined or, better yet, abandoned.

**Response:** We removed all instances of ‘simple sepsis’.

**Concern 11:** For the investigation into temporal change – did this include all individuals? This should be included in the Methods; the first mention of this should not be in Results. In examining Figure 4, it appears that the serial measurement was only performed in a subset of patients, and this is confirmed in the legend, so a description of why and how this occurred is helpful.

**Response:** The 0 and 3 hour measures were only available on a subset of patients (n=27), largely because the patient declined the second draw or was otherwise unavailable due to transfer or discharge. We have clarified this subset as patients in which their clinical course and consent allowed a 2^nd^ draw.

**Concern 12:** The statement “suggesting that blood culture misses a significant percentage of bacteremic cases” is interpretive and should be moved to the Discussion.

**Response:** Agreed. This is now deleted.

**Concern 13:** In Figure 7, specific cases are highlighted. How and why were these cases chosen? What is different about them as compared to the ones that were not included? Some mechanism to disclose this information would be appreciated.

**Response:** Agreed. We noted that these examples are chosen because they represent ‘non-synonymous’ cases of pathogen load and RNA biomarker levels. We explain more clearly that they are cases where pathogen levels and the RNA biomarkers are “highly correlated or highly disconnected”.

**Concern 14:** Throughout the manuscript, then N’s are very different in each group. For example, in Figure 6, the N’s are 20 / 20 / 19 for No Infection / Severe Sepsis / Septic Shock while in Table 1, the N’s are 24 / 26 / 22. An explanation or description of the missing patients would be helpful.

**Response:** True. The changing N’s are due to the specific criteria involved in each of the figures and reflect our best efforts to be accurate about the size of the groups in each comparison. In Figure 6, for example, we did not always have excess blood for the isolation of whole blood DNA, and thus the N is smaller. We have added text to clarify this.

**Final Issues:**

That said, there are considerable limitations given the quite restrictive inclusion criteria that were used. I would certainly welcome the opportunity to review this manuscript again after revision, because I do think, in light of the current advancement in diagnostics – including expression assays – such knowledge and understanding is helpful.

In summary, though I find the concept and results intriguing and certainly worthy of publication, in light of the contemporary definition of sepsis, I find the results, as presented, difficult to interpret. Perhaps the results would be different if it were presented as ‘no infection,’ ‘infection,’ and ‘sepsis’ by the Sepsis-3 definitions. Unfortunately, it is difficult to assess given the small sample size and heterogeneity seen in the study population vs the control group, and without great understanding of the adjudication process. In any case, the findings suggest that temporal changes in gene expression are important; thus, reformatting of the manuscript may make these results more easily interpreted in the context of our current definitions. Whether or not this is clinically meaningful is in question, but this small (though rigorous) study is meant to be hypothesis generating.
