# Supplementary material for: Blood RNA biomarkers and a point-of-care elastase assay for detecting host immune activation in suspected sepsis: Trajectory matters
Source: PLoS One. 2025 Dec 12;20(12):e0338012. doi: 10.1371/journal.pone.0338012 (PMC12700442; doi:10.1371/journal.pone.0338012)
Supplement: Attachment — Submitted filename: SENSOR Response #2.docx [file pone.0338012.s006.docx]

**Response to Editorial Concerns:** (LaFleur et al. PONE-D-25-10033R1)

**Overall:** At this stage, I have only a few minor points that should be addressed before the manuscript can proceed to acceptance. These are primarily editorial and clarificatory in nature:

**Concern:** State statistical approach in more detail.
Briefly describe normality testing, justify the use of parametric or non-parametric tests.

**Response:** A brief “Statistical Methods” paragraph was added to address this.

**Concern:** Ensure consistent figure and table formatting.
Verify that figure numbering matches the text; add missing units to all axes and continuous variables in tables.

**Response:** We verified that figure numbering matches the text. We added several units that were missing in the figures, especially Figure 4. We could not detect any missing units in the only table, Table 1.

**Concern:** Add or confirm data availability information.
Please provide a DOI or repository link for the underlying data, as required by journal policy.

**Response:** Due to the US government shutdown, it has been impossible to get an accession at Gene Expression Omnibus (GEO), and it may take months to resolve a 40 day backlog. Because there are only 6 gene expression levels per patient, we have included the patient level raw data, in anonymous form, in Supplementary Table 2.

**Concern:** Clarify the “No Sepsis” group definition.
Please explicitly define this cohort as sepsis-alert positive but non-infectious inflammatory controls to avoid confusion regarding elevated CRP and inflammatory markers.

**Response:** Correct. We inserted a sentence to this effect in the methods.

**Concern:** Expand the limitations paragraph slightly.
Explicitly acknowledge the small trajectory subset, modified enrollment criteria, and adjudication variability.

**Response:** True, these limitations were added to the end of the discussion.

**Concern:** Proofread for minor typographical and formatting inconsistencies.
(e.g., spacing, capitalization in figure labels, consistent terminology such as “flu” vs “influenza.”)

**Response:** We corrected figure labels for consistent capitalization, and made several corrections for consistent terminology.
